# Effects of Topography and PDGF on the Response of Corneal Keratocytes to Fibronectin-Coated Surfaces

**DOI:** 10.3390/jfb14040217

**Published:** 2023-04-13

**Authors:** Kevin H. Lam, Tarik Z. Shihabeddin, Jacob A. Awkal, Alex M. Najjar, Miguel Miron-Mendoza, Daniel P. Maruri, Victor D. Varner, W. Matthew Petroll, David W. Schmidtke

**Affiliations:** 1 Department of Bioengineering, University of Texas at Dallas, Richardson, TX 75080, USA; 2 Department of Ophthalmology, University of Texas Southwestern Medical Center, Dallas, TX 75390, USA; 3 Department of Surgery, University of Texas Southwestern Medical Center, Dallas, TX 75390, USA; 4 Department of Biomedical Engineering, University of Texas Southwestern Medical Center, Dallas, TX 75390, USA

**Keywords:** collagen fibrils, corneal keratocytes, wound healing, microfluidics, platelet-derived growth factor, fibronectin, topography

## Abstract

During corneal wound healing, corneal keratocytes are exposed to both biophysical and soluble cues that cause them to transform from a quiescent state to a repair phenotype. How keratocytes integrate these multiple cues simultaneously is not well understood. To investigate this process, primary rabbit corneal keratocytes were cultured on substrates patterned with aligned collagen fibrils and coated with adsorbed fibronectin. After 2 or 5 days of culture, keratocytes were fixed and stained to assess changes in cell morphology and markers of myofibroblastic activation by fluorescence microscopy. Initially, adsorbed fibronectin had an activating effect on the keratocytes as evidenced by changes in cell shape, stress fiber formation, and expression of alpha-smooth muscle actin (α-SMA). The magnitude of these effects depended upon substrate topography (i.e., flat substrate vs aligned collagen fibrils) and decreased with culture time. When keratocytes were simultaneously exposed to adsorbed fibronectin and soluble platelet-derived growth factor-BB (PDGF-BB), the cells elongated and had reduced expression of stress fibers and α-SMA. In the presence of PDGF-BB, keratocytes plated on the aligned collagen fibrils elongated in the direction of the fibrils. These results provide new information on how keratocytes respond to multiple simultaneous cues and how the anisotropic topography of aligned collagen fibrils influences keratocyte behavior.

## 1. Introduction

Normal and proper healing of the cornea is a complex process that is regulated by the local microenvironment. In the uninjured cornea, quiescent keratocytes have a dendritic morphology and are nestled between lamellae of aligned collagen fibrils [1]. Upon injury, a variety of soluble growth factors and proteins are released into the wounded area and cause the unactivated keratocytes to transform into a repair phenotype that involves differentiation into fibroblasts and myofibroblasts [2]. Some of the growth factors and proteins that have been identified to be important in corneal wound healing include fibronectin, tenascin, transforming growth factor-beta (TGF-β), fibroblast growth factor (FGF), insulin growth factor (IGF), and platelet-derived growth factor-BB (PDGF-BB) [3]. PDGF-BB is a component in tear fluid [4] which is produced by the corneal epithelium [5] and is bound at high levels to the epithelial basement membrane [6]. Upon injury to the epithelial basement membrane, large amounts of PDGF-BB are released into the corneal stroma and bind to PDGF receptors on corneal keratocytes [7]. The binding of PDGF-BB has been shown to modulate several keratocyte behaviors including proliferation and migration [8,9,10,11]. In addition, the treatment of keratocytes with PDGF induces them to adopt a more elongated morphology [11,12].

Previous studies have established that the secretion of fibronectin into the wound area is one of the first steps in the normal wound-healing response [13,14]. Fibronectin accumulation has been observed during corneal fibrosis following injury [15,16,17]. It has been shown that fibronectin can link cells to other ECM proteins such as collagen and fibrin [18], and that incorporation of fibronectin into collagen gels increased the migration rate of corneal fibroblasts [19]. Other in vitro studies have reported that TGF-β-induced myofibroblast transformation of corneal keratocytes requires fibronectin fibril formation [20,21]. Using 3D fibrin matrices, corneal fibroblasts were shown to secrete and organize fibronectin into tracks that are used by other trailing cells as migratory conduits to form interconnected lines of cells [22,23]. Similarly, in vivo studies have demonstrated the importance of fibronectin in promoting cell adhesion and migration in the cornea following injury [20], and that topical administration of fibronectin facilities corneal epithelial wound healing [24].

ECM proteins such as type I collagen [25,26,27] and fibronectin [28,29] can bind many growth factors directly or in combination with heparin and heparin sulfate [30]. Studies have shown that PDGF-BB binds to fibronectin [31,32] as well as to both immobilized type I collagen as well as type I collagen fibrils reconstituted in vitro [25]. Moreover, the binding of secreted molecules to the ECM can regulate the bioavailability of the molecules, where the ECM acts as a local reservoir of these molecules and protects them from degradation [30,33]. In addition, several studies have noted that the extracellular matrix (ECM) not only influences cell behavior by providing structural support and topographical cues, but it can also work in concert with secreted growth factors, cytokines, and proteins to regulate cell behavior. For example, it has been reported that migration of human dermal fibroblasts is controlled by distinct and overlapping signaling pathways related to PDGF-BB and collagen binding [34]. Likewise myofibroblast differentiation of rabbit corneal keratocytes has been shown to require synergistic integrin and growth factor signaling involving the fibronectin receptor, TGF-β, and PDGF [35].

Previously, we have developed a microfluidic patterning technique that generates aligned collagen fibrils that are similar to the structure of the native corneal stroma [36]. Using these substrates, we investigated how corneal keratocytes respond to the simultaneous exposure of topographical cues (i.e., aligned collagen fibrils) and soluble biochemical cues (PDGF-BB and TGFβ) [37,38]. Given the importance of the interactions between secreted molecules and the ECM in corneal wound healing, the present study describes the behavior of primary rabbit keratocytes when cultured on substrates in the presence of adsorbed fibronectin alone and in combination with biophysical (aligned collagen) and/or soluble (PDGF-BB) cues. 

## 2. Materials and Methods

### 2.1. Fabrication of Aligned Collagen Fibrils

Aligned collagen fibrils were deposited onto glass coverslips by perfusing chilled solutions of Type I collagen at well-defined shear rates through straight microfluidic channels placed on a hotplate as previously described [36,38]. Briefly, poly(dimethylsiloxane) (PDMS) microfluidic stamps were exposed to an air plasma (Harrick Plasma, Ithaca, NY, USA) for 1 min on high RF before they were sealed to hydrophobic glass coverslips that had been previously cleaned for 1 hr with a 28% solution of nitric acid and then functionalized with a 1% AquaSil siliconizing solution for 15 s. The microfluidic devices were then placed on a digital hotplate (Torrey Pines Scientific, Carlsbad, CA, USA), with a surface temperature of 37 °C in a cold room (4 °C) for 30 min prior to perfusion of the collagen solution through the microfluidic channel. The collagen solution (1.6 mg/mL) was made by mixing a ratio of 8 parts collagen (Advanced Biomatrix, Carlsbad, CA, USA), 1 part 10X MEM (Gibco, Waltham, MA, USA), and 1 part 0.1 M NaOH. Additional 1X MEM (Gibco) was added to the solution to bring the collagen concentration to the desired amount, and the solution was kept on ice to prevent premature collagen polymerization in the tube. The pH of the solution was adjusted to between 7.2 and 7.6 with the addition of NaOH. Initially the collagen solution was perfused at a rate of 10 μL/min for 4 min, after which, the infusion rate was reduced to 7.5 μL/min (shear rate = 150 s^−1^) for 30 min. After collagen perfusion, the microfluidic stamps were peeled from the glass coverslips, rinsed with Millipore water, and allowed to dry on the hot plate (37 °C) for at least 30 min before being placed into Fluoroware holders (FSI International; Chaska, MN, USA) until needed.

### 2.2. Fabrication and Coating of PDMS Ring—Microwell Culture System

**Figure 1:**
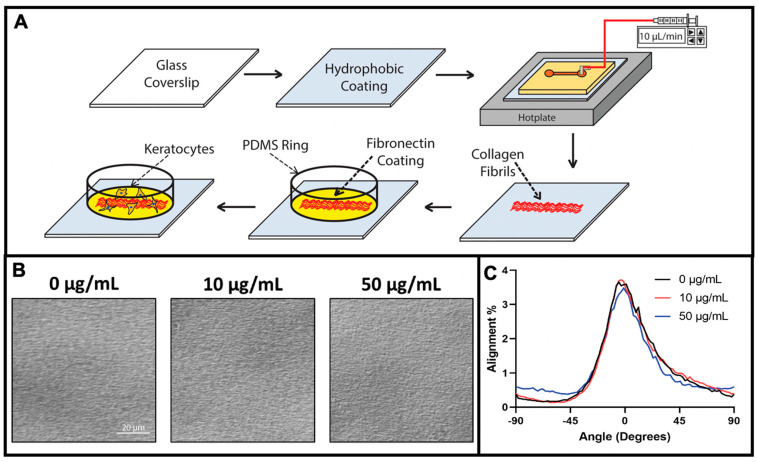
Schematic illustration of collagen fibril fabrication, protein modification, and cell culture. (**A**) A clean glass coverslip is coated with a hydrophobic coating of aquasil and incorporated as the bottom wall of a straight channel microfluidic device. The microfluidic device is placed on a hotplate and infused with a chilled collagen solution. As the collagen solution is perfused through the microchannel it polymerizes and aligned collagen fibrils are deposited on the hydrophobic surface. The microfluidic device is peeled off the substrate, and a PDMS ring is placed on the fibril-coated substrate to serve a cell culture well. Subsequently, the surface is modified with a coating of fibronectin, and keratocytes are seeded and cultured. (**B**) Representative differential interference contrast (DIC) microscopic images of aligned collagen fibrils after coating with 0, 10, or 50 μg/mL fibronectin solution. (**C**) Quantitative analysis of the alignment of the collagen fibrils in panel (**B**).

Glass coverslips coated with aligned collagen fibrils were assembled into a PDMS ring-glass coverslip culture system to allow for high-resolution microscopic imaging, as previously described [36,38]. Briefly, a PDMS ring (inner diameter = 15 mm, outer diameter = 30 mm) was treated with a high RF air plasma for 1 min (Harrick) and the PDMS ring was immediately brought into contact with the collagen fibril-coated glass coverslip to form a permanent seal and creating a well for cell culture (Figure 1).

To investigate the effects of fibronectin on keratocyte behavior, solutions of different fibronectin concentrations (1–50 μg/mL) were pipetted into the PDMS ring-glass coverslip culture devices and allowed to incubate for 2 h at room temperature. The fibronectin solutions were prepared by diluting a stock solution of either an unlabeled fibronectin (EMD Millipore) or HyLite 488-tagged fibronectin (CytoSkeleton; San Diego, CA, USA) with phosphate-buffered saline (PBS) to the desired concentration. Following the 2 h incubation, the fibronectin solution was aspirated from the culture device and the devices were washed with PBS before cell seeding [39].

### 2.3. Primary Keratocyte Extraction and Cell Culture

Primary corneal keratocytes (NRKs) were isolated from the eyeballs of New Zealand white rabbits (Pel-Freez Biologicals; Rogers, AR, USA) and cultured in basal serum-free media containing Dulbecco’s modified Eagle’s medium (DMEM) that was supplemented with 100 μM non-essential amino acids (Invitrogen; Carlsbad, CA, USA), 100 μg/mL ascorbic acid, 1% RPMI vitamin mix, and 1% PenStrep (Invitrogen; Carlsbad, CA, USA) as previously described [36,38]. After 4 days, NRK cells were seeded in the PDMS ring-glass coverslip culture devices at a density of 20,000 cells/mL in 2 mL of serum-free media. In some experiments, platelet-derived growth factor (PDGF-BB) (Gibco; Waltham, MA, USA) was added to the media at a final concentration of 50 ng/mL [36,38].

### 2.4. Immunohistochemistry

After 2 or 5 days of culture, cells were fixed using a 3% paraformaldehyde solution in PBS for 15 min at room temperature and washed three times for 20 min each with PBS. To label cells for nuclei, F-actin, and α-SMA, cells were permeabilized by treatment with 0.5% triton X-100 for 15 min, washed for 10 min with PBS, blocked for 1 hr at room temperature with 1% bovine serum albumin (BSA, fraction V) in PBS, and washed with PBS for 20 min three additional times. Cells were stained for α-SMA by incubation for 2 h at 37 °C with primary mouse anti-human α-SMA antibody (Sigma-Aldrich, St. Louis, MI, USA; 1:600 dilution), washed 3 times with PBS (20 min/wash), and incubated for 1 h with FITC conjugated goat anti-mouse secondary antibody (1:200) (Jackson Immunoresearch; West Grove, PA, USA) and/or Alexa-Fluor 564 Phalloidin (1:1000 in DMSO) at 37 °C. Samples were subsequently washed 3 times with PBS (20 min/wash). To label cell nuclei, cells were stained with DAPI (Life Tech, Carlsbad, CA, USA) for 20 min, and washed with PBS. Samples were imaged with a Zeiss AxioObserver Inverted Microscope using a 20X Plan-Apochromat (NA = 0.8) and/or a 63X Plan-NeoFluor (NA = 1.4) objective. To determine the percentage of αSMA-positive cells, the number of αSMA-positive cells was counted for each condition and divided by the total number of cells. Fluorescent images from six different regions of interest were taken at random for each sample, three regions of cells adhered to collagen fibrils, and three regions of cells adhered to the glass. Each condition was evaluated in at least 3 independent experiments.

### 2.5. Keratocyte Alignment

The degree of keratocyte alignment was measured using an ImageJ Directionality plugin that uses a Fourier component analysis procedure, and then calculating an orientation index (OI) [36,38]. An OI value of 100% indicates keratocyte co-alignment in the direction of the aligned collagen fibrils, 0% represents random keratocyte alignment, and 100% indicates keratocyte alignment perpendicular to the fibril direction.

### 2.6. Statistical Analysis

GraphPad Prism (GraphPad; San Diego, CA, USA) was used to for statistical analysis. A three-way ANOVA with a Holm–Sidak post hoc test was used for comparing differences in the percentage of α-SMA cells and keratocyte alignment.

## 3. Results and Discussion

### 3.1. Fabrication of Fibronectin Coated Aligned Collagen Fibrils

Collagen type I fibrils make up a majority of the native corneal stroma, and the aligned lamellar organization of these fibrils is a key feature of the ECM. In addition to providing topographical cues, fibrillar collagen has binding sites for interactions with nearly 50 other molecules [40], some of which have important roles in mediating keratocyte behavior. Although several reports have suggested an interplay between collagen fiber topography and ECM proteins in modulating keratocyte adhesion, migration, and differentiation, our understanding of this interdependence is limited [41,42,43]. To determine the interplay between the topographical cues that aligned collagen fibrils provided and the effects of specific ECM proteins on keratocyte behavior, we have developed a method to fabricate substrates with aligned collagen fibrils that are subsequently modified with other ECM proteins. Type I collagen fibrils were fabricated on glass coverslips by a multi-step procedure as previously described, and then coated with fibronectin (Figure 1). We have chosen plasma fibronectin as a representative ECM protein for several reasons. First, fibronectin contains numerous RGD sequences which is a principle binding domain for a number of integrins (e.g., α_5_β_1,_ α_4_β_1_, α_6_β_1_, α_V_β_1_, α_V_β_6_) [44]. In addition, fibronectin has been reported to bind to fibrillar collagen, and is present in the corneal stroma following an injury [45,46]. Finally, it has been reported that corneal epithelial cells migrate in vitro in response to both soluble (chemotaxis) and bound (haptotaxis) fibronectin [47]. Figure 1B shows representative images of aligned collagen fibrils that had been coated with different fibronectin solutions. To quantify the degree of collagen fibril alignment we measured the fibril directionality with an ImageJ Directionality plug-in to generate alignment histograms. As shown in Figure 1C, coating the aligned collagen fibrils had no effect on fibril alignment.

### 3.2. Effect of Topographical and ECM Cues on Keratocyte Behavior in the Absence of Growth Factors

**Figure 2:**
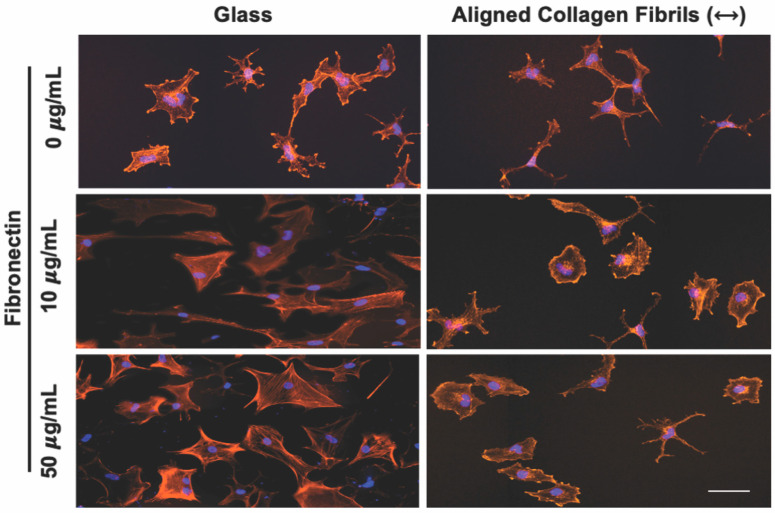
Influence of fibronectin coating on corneal keratocyte morphology after 2 days of culture. Representative fluorescent images of corneal keratocytes cultured in serum-free media for 2 days on glass or aligned collagen fibrils coated with different fibronectin concentrations (0, 10, or 50 µg/mL). Scale = 100 µm. The aligned collagen fibrils were patterned horizontally. Cells were stained for F-actin (orange) and DAPI (blue).

To determine how keratocytes respond to simultaneous exposure to ECM compositional and topographical cues, we seeded normal rabbit corneal keratocytes (NRKs) in serum-free media for 2 or 5 days on top of fibronectin-coated substrates that contained regions of aquasil-coated glass and regions of aligned collagen fibrils. To visualize changes in the morphology and activation of the cultured keratocytes, cells were fixed, and fluorescently labeled for nuclear DNA (DAPI), F-Actin, and α-SMA. When keratocytes were cultured for 2 days, cells adhering to the glass regions coated with fibronectin (10 or 50 μg/mL) exhibited visible stress fibers and had a broad polygonal morphology (Figure 2). In contrast, when cells were cultured on glass substrates coated with a lower concentration of fibronectin (5 μg/mL) or in the absence of fibronectin (Figure 2), the keratocytes displayed a stellate morphology with no visible stress fibers.

When keratocytes were cultured in serum-free media for 2 days on aligned collagen fibrils without a fibronectin coating they displayed a dendritic morphology, and did not express visible stress fibers. In contrast, keratocytes cultured on fibronectin-coated aligned collagen fibrils (10 or 50 μg/mL fibronectin) displayed a variety of morphologies that were different than those observed on the fibronectin-coated glass regions (Figure 2). Some keratocytes had a stellate morphology, whereas others displayed a spread morphology with striations resembling stress fibers. We did not observe any noticeable difference in the morphologies of keratocytes cultured on the 10 or 50 μg/mL fibronectin-coated aligned collagen fibrils. We also did not observe any preferential alignment of the keratocytes in the direction of the aligned collagen fibrils.

**Figure 3:**
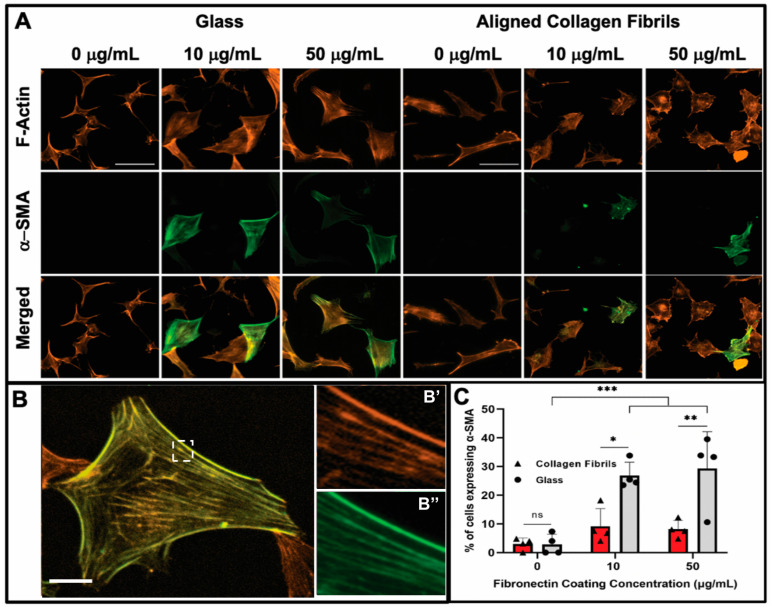
Influence of fibronectin coating and aligned collagen fibrils on α-SMA expression levels after 2 days of culture. (**A**) Representative fluorescent images of corneal keratocytes cultured in serum-free media for 2 days on glass or aligned collagen fibrils coated with different fibronectin concentrations (0, 10, or 50 µg/mL). Cells were stained for F-actin (orange) and α-SMA (green), scale = 100 µm. The aligned collagen fibrils were patterned horizontally. (**B**) Zoomed image of a single cell, with the dashed white box indicating insets (**B’**,**B’’**) which show the co-localization of F-actin and α-SMA respectively, scale = 25 µm. (**C**) Plot of the percentage of cells positive for α-SMA immunofluorescence on glass and aligned collagen fibrils as a function of fibronectin coating concentration. Mean ± s.d. is shown for 4 experimental replicates. A two-way ANOVA with a Tukey post hoc test was used to evaluate the significance between groups (* *p* < 0.05; ** *p* < 0.01; *** *p* < 0.001; n.s., not significant). Reproduced and adapted from reference [39].

The observed differences between keratocyte behavior on the fibronectin-coated glass and aligned collagen fibrils led us to conduct a second set of experiments in which we assessed whether keratocytes were undergoing myofibroblastic transformation by staining for α-SMA expression. As shown in Figure 3, we observed that a significant number of keratocytes (~27% and ~29%) cultured for 2 days on the glass regions coated with 10 or 50 μg/mL fibronectin were positive for α-SMA immunofluorescence (Figure 3C), whereas keratocytes adhered to the aquasil-coated glass showed minimal α-SMA expression. Conversely, less than ~10% of the keratocytes on the fibronectin-coated aligned collagen fibrils displayed α-SMA immunofluorescence after 2 days.

**Figure 4:**
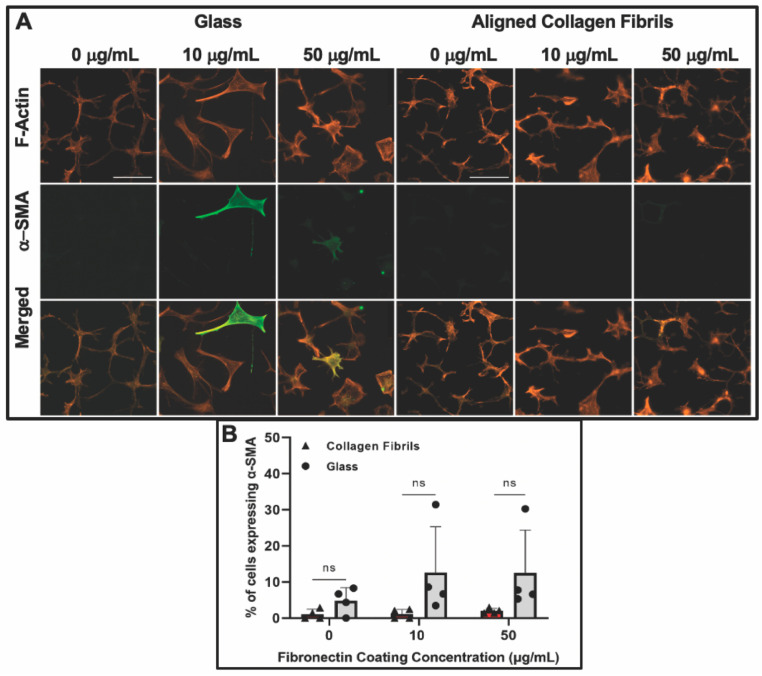
Influence of fibronectin coating and aligned collagen fibrils on α-SMA expression levels after 5 days of culture. (**A**) Representative fluorescent images of corneal keratocytes cultured in serum-free media for 5 days on glass or aligned collagen fibrils coated with different fibronectin concentrations (0, 10, or 50 µg/mL). Cells were stained for F-actin (orange) and α-SMA (green), scale = 100 µm. The aligned collagen fibrils were patterned horizontally. (**B**) Plot of the percentage of cells positive for α-SMA immunofluorescence on glass and aligned collagen fibrils as a function of fibronectin coating concentration. Mean ± s.d. for 4 experimental replicates. A two-way ANOVA with a Tukey post hoc test was used to evaluate significance between groups. (n.s., not significant). Reproduced and adapted from reference [39].

After day 5 of culture, some keratocytes on the fibronectin-coated glass maintained a spread morphology; however, other keratocytes appeared to revert back to a more quiescent state as indicated by a stellate morphology with dendritic processes (Figure 4). The percentage of α-SMA positive keratocytes on the fibronectin-coated glass dropped to ~13% and ~12% for 10 and 50 µg/mL, respectively, after 5 days of culture (Figure 4B). A similar trend of keratocytes reverting back to a more quiescent state was observed for keratocytes adhered to the fibronectin-coated collagen fibrils (Figure 4) with only ~1% (10 µg/mL) and ~2% (50 µg/mL) of the cells expressing α-SMA after 5 days of culture (Figure 4B). Our observation that keratocytes cultured on flat fibronectin-coated surfaces have increased levels of α-SMA expression is in agreement with previous studies with human lung fibroblasts [48], porcine lens epithelial cells [49], and human retinal pigment epithelial cells [50]. Taken together, these results suggest that the extent to which adsorbed fibronectin causes activation of keratocytes depends on whether they are cultured on a rigid flat surface such as glass or a more natural substrate with topographical cues such as aligned collagen fibrils. It is interesting to note that in contrast to our previous studies [36,38] in which keratocytes aligned in the presence of a growth factor, such as PDGF-BB or TGFβ, the cells did not align in the direction of the fibrils in either the presence or absence of fibronectin when cultured in basal serum-free media. At this time, the exact reason(s) for why a reduced number of cells become activated (i.e., reduced stress fiber and α-SMA expression) on the aligned collagen fibrils when exposed to adsorbed fibronectin is unknown. Previously, it has been reported that topography can inhibit the myofibroblastic transformation of keratocytes and that keratocytes will undergo a negative feedback loop and revert back to their quiescent state if seeded on aligned fibrils [51,52,53]. Thus, the topography of the aligned collagen fibrils may be playing an important role in modulating the morphology of the keratocytes. However, we cannot completely rule out the possibility that the amount of fibronectin adsorbed to the aligned collagen fibrils is lower than the amount of fibronectin adsorbed to the glass. The fact that no difference was observed in the number of α-SMA positive cells on the aligned collagen fibrils coated with 10 and 50 μg/mL fibronectin would argue against differing amounts of adsorbed fibronectin being the cause (Figure 3C).

At this time it is unknown why the keratocytes appeared to return to a more quiescent state at day 5 on the fibronectin-coated substrates (e.g., glass and aligned collagen fibrils). One possibility is that the keratocytes are degrading the fibronectin and depositing their own ECM over time. Alternatively, it could be that the fibronectin is slowly desorbing from the surface with time since our coating procedure does not covalently bind the fibronectin to either the glass surface or the aligned collagen fibrils. Thus, at later time points there may be insufficient fibronectin adsorbed to maintain the keratocytes in an activated state. To investigate the stability of the fibronectin coating as a function of time under typical cell culture conditions (i.e., 37 °C, serum-free media), we initially coated aquasil-coated glass and aligned collagen fibrils with multiple drops of a 10 μg/mL HiLyte Fluor™ 488 labeled fibronectin solution and changes in the fluorescent signal were optically measured over time (Supplementary Figure S1). The fluorescent signal for each drop was only measured once at a defined time point in order to prevent any possible photobleaching that might occur if multiple exposures to the excitation light were performed. During the first 24 h of exposure to cell-culture conditions, a significant drop (~48–50%) in the fluorescent intensity occurred, whereas during the next 24 h (48 h after the initial incubation), there was a modest drop (~12–15%) in the fluorescent level on both fibrils and glass. Measurement of the fluorescent levels after 5 days in culture showed little difference (3–7%) from the 48 hr levels. At this time, the exact nature of the drop between 0 and 24 h is unknown but is probably due to simple protein desorption. Alternatively, this drop may be due to the degradation of the HiLyte 488 fluorescent tag. To determine whether degradation of the fluorescent tag was the cause, we performed a second set of experiments in which we (i) coated aquasil-coated glass slides with a 10 μg/mL fibronectin solution made with the non-labeled fibronectin, (ii) incubated the fibronectin-coated slides in a PBS solution at 37 °C for 0–5 days, and (iii) immunostained the fibronectin-coated slides with a primary antibody to fibronectin, followed by an Alexa-647 labeled secondary antibody. Similar to the results of Figure S1, we saw a large drop (~57%) after the first 24 h, followed by smaller drops at 2 and 5 days (Supplementary Figure S2). These results suggest that the drop in adsorbed fibronectin that we observed was not necessarily due to the degradation of the fluorescent tag.

To further examine whether fibronectin desorption may be the cause of the decrease in keratocyte expression of α-SMA with time, we conducted an additional set of experiments in which substrates coated with 10 and 50 µg/mL fibronectin were incubated in cell culture media in a CO_2_ incubator at 37 °C for two days to allow for fibronectin desorption to occur before keratocytes were seeded and cultured on them. When keratocytes were cultured on these “desorption” substrates, we observed results similar to those in Figure 2, Figure 3 and Figure 4. After 2 days of culture on the “desorption” substrates, keratocytes displayed a broad polygonal morphology with visible stress fibers and had similar levels of α-SMA immunofluorescence as keratocytes cultured on “no desorption” substrates (Supplementary Figure S3A). After 5 days of culture on the “desorption” substrates, we once again observed the keratocytes display a more quiescent morphology and lower levels of α-SMA expression (Supplementary Figure S3B). The fact that most of the fibronectin desorption occurs during the first 48 h (Figures S1 and S2) whereas keratocytes still became activated on the preconditioned “desorption” substrates suggests that there was a sufficient amount of fibronectin retained on the substrate to cause activation of keratocytes. Thus, these results suggest that the drop in keratocyte activation is not solely due to fibronectin desorption. The exact cause of the decrease in keratocyte activation is under further study.

### 3.3. Keratocyte Response to ECM Composition and Topographical Cues in the Presence of PDGF-BB

**Figure 5:**
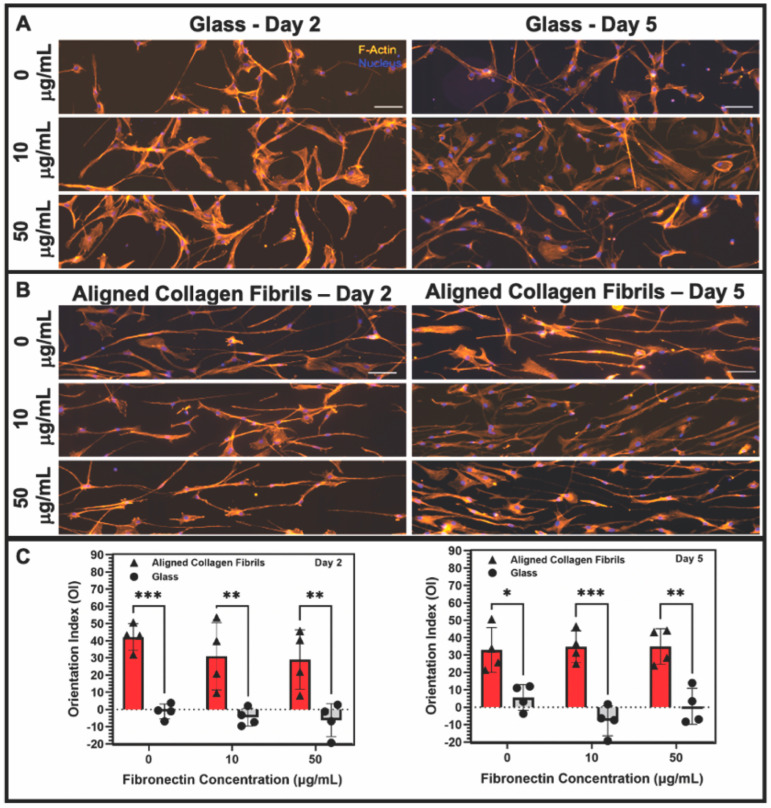
Influence of soluble PDGF-BB on keratocyte response to fibronectin coatings. Representative fluorescent images of keratocytes cultured for 2 or 5 days in the presence of 50 ng/mL PDGF-BB in solution on (**A**) glass or (**B**) aligned collagen fibrils coated with different concentrations of fibronectin. Cells were stained for F-Actin (orange) and Nucleus (DAPI), scale = 100 µm. The aligned collagen fibrils were patterned horizontally. (**C**) Plot of the orientation index values for the alignment of keratocytes cultured on fibronectin-coated glass or fibrils at days 2 and 5 in the presence of PDGF-BB. (Mean ± s.d. is shown for 4 experimental replicates). A two-way ANOVA with a Tukey post hoc test was used to evaluate the significance between groups (* *p* < 0.05; ** *p* < 0.01; *** *p* < 0.001). Reproduced and adapted from reference [39].

Platelet-derived growth factor (PDGF) is a powerful cytokine released by corneal epithelial cells and present in tears following corneal injury. PDGF stimulates keratocyte proliferation and chemotaxis, and modulates the transformation of quiescent keratocytes into fibroblasts [54,55,56]. Thus, we investigated how soluble PDGF-BB would influence the response of keratocytes to ECM and topographical cues. Similar to the experiments described previously, keratocytes were cultured for 2 to 5 days on glass and aligned collagen fibrils coated with different amounts of fibronectin but in serum-free media supplemented with PDGF-BB. Keratocytes cultured for 2 days on fibronectin-coated glass had a narrow, elongated morphology with multiple randomly oriented extensions (Figure 5A). When PDGF-BB was present in the media very few, if any, keratocytes displayed stress fibers on the fibronectin-coated substrates (10 or 50 μg fibronectin) or aquasil-coated glass control. Similarly, keratocytes that were cultured for 5 days in the presence of PDGF-BB on fibronectin-coated glass also displayed an elongated morphology (Figure 5A).

When keratocytes were cultured on aligned collagen fibrils coated with fibronectin for 2 or 5 days in the presence of PDGF-BB, they displayed an elongated morphology that was in alignment with the direction of the collagen fibrils (Figure 5B). We confirmed the suitability of using fluorescent images of the actin cytoskeleton to be representative of the shape of keratocytes by comparing phase contrast images to the fluorescent images. As shown in Supplementary Figure S4, the shape of keratocytes was captured by the fluorescent images. Qualitatively, there were no clear differences in the morphology of keratocytes adhered to uncoated or fibronectin-coated collagen fibrils. To quantitatively assess any differences in the orientation of the keratocytes cultured in the presence of PDGF-BB on fibronectin-coated glass or aligned collagen fibrils, we measured the orientation index (OI). An OI value of 0% is representative of cells having a random alignment, an OI value of 100% indicates parallel alignment of the cells and the collagen fibrils, whereas an OI value of −100% indicates perpendicular alignment between the cells and fibrils. Keratocytes that were cultured on fibronectin-coated glass substrates in the presence of PDGF-BB had a random cell alignment as assessed by their OI values being slightly negative (2 days) or close to zero (5 days). In contrast, keratocytes that were cultured for 2 days in the presence of PDGF-BB on either uncoated aligned collagen fibrils (OI = 45%) or fibronectin-coated fibrils (OI = 30%) showed an increased degree of alignment. (Supplementary Figure S5 shows representative phase contrast images in which the alignment of collagen fibrils and keratocytes can be directly visualized). Likewise, keratocytes cultured for 5 days in the presence of PDGF-BB on both the uncoated and fibronectin-coated collagen fibrils remained aligned (OI ~30%). A statistical analysis of the OI values showed that the degree of alignment of keratocytes cultured on aligned collagen fibrils was significantly higher than on glass (Figure 5C). However, there was no statistically significant difference in the level of alignment of keratocytes cultured on uncoated or fibronectin-coated collagen fibrils at 2 or 5 days (*p* > 0.05). The quantitative data in Figure 5C confirm the qualitative results (Figure 5B) and demonstrate that keratocytes cultured on aligned collagen fibrils in the presence of PDGF-BB become aligned and elongated in the direction of the collagen fibrils, in agreement with our prior studies [36,38]. The fact that the absence or presence of adsorbed fibronectin had little to no effect on keratocyte alignment suggests that ECM topography in combination with PDGF-BB was a more powerful cue than ECM composition.

**Figure 6:**
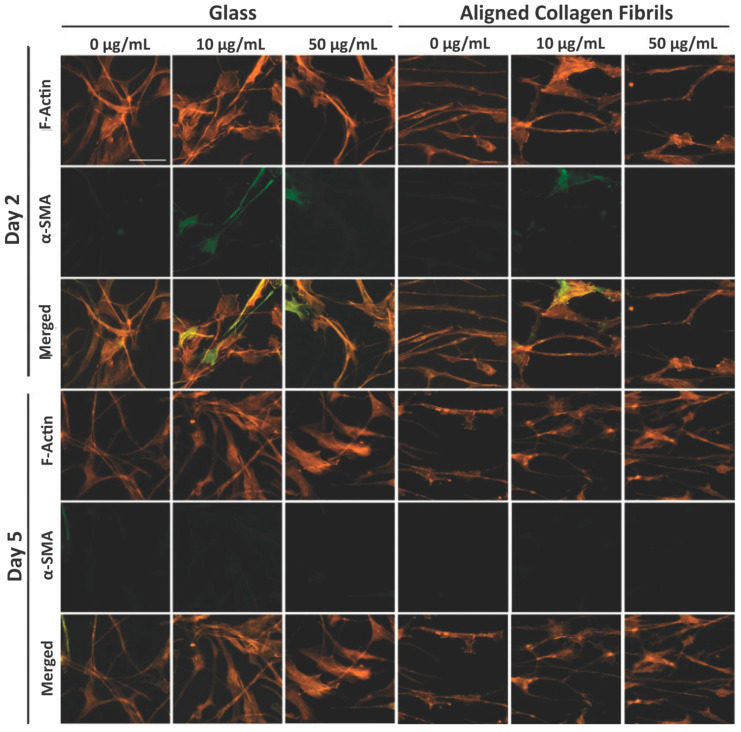
Influence of soluble PDGF-BB on α-SMA expression levels. Representative fluorescent images of corneal keratocytes cultured in the presence of 50 ng/mL PDGF-BB in solution for 2 and 5 days on glass or aligned collagen fibrils coated with different fibronectin concentrations (0, 10, or 50 µg/mL). Cells were stained for F-actin (orange) and DAPI (blue), scale = 100 µm. The aligned collagen fibrils were patterned horizontally. Reproduced and adapted from reference [39].

**Figure 7:**
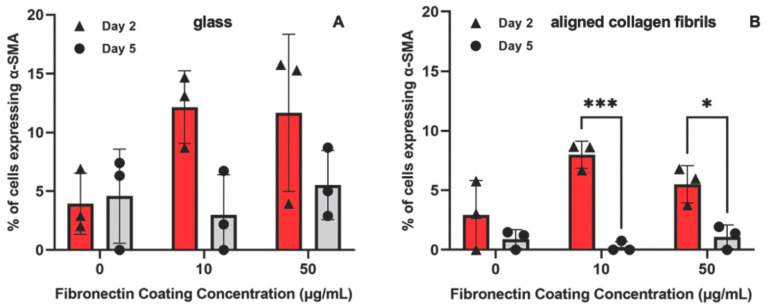
Effect of substrate topography and culture time on α-SMA expression levels. Plot of the percentage of cells positive for α-SMA immunofluorescence on (**A**) glass and (**B**) aligned collagen fibrils as a function of fibronectin coating concentration when corneal keratocytes were cultured in the presence of 50 ng/mL PDGF-BB for 2 and 5 days. Mean ± s.d. for 3 experimental replicates. A two-way ANOVA with a Tukey post hoc test was used to evaluate the significance between groups (* *p* < 0.05; *** *p* < 0.001).

To determine whether the presence of PDGF-BB-induced keratocytes to undergo myofibroblastic activation, we also stained the cells for α-SMA (Figure 6). Approximately ~12% of the keratocytes cultured in the presence of PDGF-BB on fibronectin-coated glass for 2 days expressed α-SMA (Figure 7A). However, after 5 days, the percentage of α-SMA positive keratocytes cultured on either the 10 or 50 µg/mL fibronectin-coated glass dropped to control levels (<5%). Keratocytes that were cultured on fibronectin-coated aligned collagen fibrils in the presence of PDGF-BB had lower levels of α-SMA expression as compared to glass at 2 days of culture and essentially no α-SMA expression at 5 days (Figure 7B). Although these data are from two different experiments, it is interesting to note that the α-SMA expression of keratocytes cultured on fibronectin-coated glass for 2 days was lower in the presence of PDGF-BB (~12%, Figure 7A) than in its absence (~25%, Figure 4C). In contrast, the level of α-SMA expression was similar (>10%) for keratocytes cultured for 2 days on aligned collagen fibrils coated with fibronectin in the presence or absence of PDGF-BB.

## 4. Conclusions

Accumulating evidence demonstrates that keratocyte behavior in vivo is influenced by multiple biochemical and biophysical cues such as topography, ECM composition, and soluble cues. Thus, in vitro platforms are needed that present multiple cues simultaneously but that allow for these cues to be manipulated independently. In this study we report a novel cell culture platform that combines several cues—topography (aligned collagen fibrils), ECM composition (fibronectin vs collagen), and soluble factors (PDGF vs serum-free media)—relevant to understanding how corneal keratocytes integrate multiple cues simultaneously. Our study demonstrated that exposure of keratocytes to adsorbed fibronectin transiently induced them to become activated as indicated by a change in their morphology and α-SMA expression. The extent of keratocyte activation was dependent not only on the length of cell culture (2 vs. 5 days) but also on whether the keratocytes were cultured (i) on aligned collagen fibrils or on a flat rigid substrate, and (ii) in the presence of PDGF or serum-free media. We also observed that PDGF was a potent cue to induce keratocytes to elongate and align in the direction of the aligned collagen fibrils. It is clear from the findings discussed here that matrix topography and composition can influence keratocyte behavior, and may have implications in the design of biomaterials utilizing fibronectin for corneal implants (e.g., corneal grafting, keratoprosthesis). We anticipate that the versatile platform described here will facilitate studies with a variety of growth factors (e.g., FGF, IGF, and TGF-β), ECM proteins (e.g., Collagen III, IV, and tenascin), and proteoglycans (e.g., lumican, keratocan, and decorin) that are intrinsic to the normal cornea stroma and/or present during wound healing. 

## Data Availability

The data presented in this study are available on request from the corresponding author.

## Supplementary Materials

The following supporting information can be downloaded at: https://www.mdpi.com/article/10.3390/jfb14040217/s1, Figure S1: Stability of fluorescent fibronectin coating; Figure S2: Stability of non-fluorescent fibronectin coating; Figure S3: Keratocyte deactivation with time on preconditioned fibronectin-coated substrates; Figure S4: Comparison of fluorescent and phase contrast images in evaluating keratocyte shape; Figure S5: Phase contrast images of keratocyte and collagen fibril alignment.

## References

[1] Hassell J.R., Birk D.E. (2010). The molecular basis of corneal transparency. Exp. Eye Res..

[2] Ljubimov A.V., Saghizadeh M. (2015). Progress in corneal wound healing. Prog. Retin. Eye Res..

[3] Yu F.S.X., Yin J., Xu K., Huang J. (2010). Growth factors and corneal epithelial wound healing. Brain Res. Bull..

[4] Vesaluoma M., Teppo A.M., Grönhagen-Riska C., Tervo T. (1997). Platelet-derived growth factor-BB (PDGF-BB) in tear fluid: A potential modulator of corneal wound healing following photorefractive keratectomy. Curr. Eye Res..

[5] Kim W., Mohan R.R., Mohan R.R., Wilson S.E. (1999). Effect of PDGF, IL-1a, and BMP2/4 on corneal fibroblast chemotaxis: Expression of the platelet-derived growth factor system in the cornea. Investig. Ophthalmol. Vis. Sci..

[6] Wilson S.E., Mohan R.R., Mohan R.R., Ambrósio R., Hong J.W., Lee J.S. (2001). The corneal wound healing response: Cytokine-mediated interaction of the epithelium, stroma, and inflammatory cells. Prog. Retin. Eye Res..

[7] Kamiyama K., Iguchi I., Wang X., Imanishi J. (1998). Effects of PDGF on the migration of rabbit corneal fibroblasts and epithelial cells. Cornea.

[8] Denk P.O., Knorr M. (1997). The in vitro effect of platelet-derived growth factor isoforms on the proliferation of bovine corneal stromal fibroblasts depends on cell density. Graefe’s Arch. Clin. Exp. Ophthalmol..

[9] Andresen J.L., Ehlers N. (1998). Chemotaxis of human keratocytes is increased by platelet-derived growth factor-BB, epidermal growth factor, transforming growth factor-alpha, acidic fibroblast growth factor, insulin-like growth factor-I, and transforming growth factor-beta. Curr. Eye Res..

[10] Iyer K.S., Maruri D.P., Peak K.E., Schmidtke D.W., Petroll W.M., Varner V.D. (2022). ECM stifness modulates the proliferation but not the motility of primary corneal keratocytes in response to PDGF-BB. Exp. Eye Res..

[11] Kim A., Lakshman N., Karamichos D., Matthew Petroll W. (2010). Growth factor regulation of corneal keratocyte differentiation and migration in compressed collagen matrices. Investig. Ophthalmol. Vis. Sci..

[12] Jester J.V., Ho-Chang J. (2003). Modulation of cultured keratocyte phenotype by growth factors/cytokines control in vitro contractility and extracellular matrix contraction. Exp. Eye Res..

[13] Wachtlin J., Langenbeck K., Schründer S., Zhang E., Hoffmann F. (1999). Immunohistology of corneal wound healing after photorefractive keratectomy and laser in situ keratomileusis. J. Refract. Surg..

[14] van Setten G.B., Koch J.W., Tervo K., Lang G.K., Tervo T., Naumann G.O.H., Kolkmeier J., Virtanen I., Tarkkanen A. (1992). Expression of tenascin and fibronectin in the rabbit cornea after excimer laser surgery. Graefe’s Arch. Clin. Exp. Ophthalmol..

[15] Kivanany P.B., Grose K.C., Petroll W.M. (2016). Temporal and spatial analysis of stromal cell and extracellular matrix patterning following lamellar keratectomy. Exp. Eye Res..

[16] Jester J.V., Petroll W.M., Cavanagh H.D. (1999). Corneal stromal wound healing in refractive surgery: The role of myofibroblasts. Prog. Retin. Eye Res..

[17] Jester J.V., Barry-Lane P.A., Petroll W.M., Olsen D.R., Cavanagh H.D. (1997). Inhibition of corneal fibrosis by topical application of blocking antibodies to TGF(β) in the rabbit. Cornea.

[18] Singh P., Carraher C., Schwarzbauer J.E. (2010). Assembly of fibronectin extracellular matrix. Annu. Rev. Cell Dev. Biol..

[19] Andresen J.L., Ledet T., Hager H., Josephsen K., Ehlers N. (2000). The influence of corneal stromal matrix proteins on the migration of human corneal fibroblasts. Exp.Eye Res..

[20] Jester J.V., Huang J., Barry-Lane P.A., Kao W.W.Y., Petroll W.M., Cavanagh H.D. (1999). Transforming growth factor(beta)-mediated corneal myofibroblast differentiation requires actin and fibronectin assembly. Investig. Ophthalmol. Vis. Sci..

[21] Sandbo N., Dulin N. (2011). Actin cytoskeleton in myofibroblast differentiation: Ultrastructure defining form and driving function. Transl. Res..

[22] Miron-Mendoza M., Graham E., Manohar S., Petroll W.M. (2017). Fibroblast-fibronectin patterning and network formation in 3D fibrin matrices. Matrix Biol..

[23] Miron-Mendoza M., Lin X., Ma L., Ririe P., Petroll W.M. (2012). Individual versus collective fibroblast spreading and migration: Regulation by matrix composition in 3D culture. Exp. Eye Res..

[24] Nishida T., Nakagawa S., Nishibayashi C., Tanaka H., Manabe R. (1984). Fibronectin Enhancement of Corneal Epithelial Wound Healing of Rabbits in Vivo. Arch. Ophthalmol..

[25] Somasundaram R., Schuppan D. (1996). Type I, II, III, IV, V, and VI Collagens Serve as Extracellular Ligands for the Isoforms of Platelet-derived Growth Factor (AA, BB, and AB). J. Biol. Chem..

[26] Somasundaram R., Ruehl M., Tiling N., Ackermann R., Schmid M., Riecken E.O., Schuppan D. (2000). Collagens serve as an extracellular store of bioactive interleukin 2. J. Biol. Chem..

[27] Schuppan D., Schmid M., Somasundaram R., Ackermann R., Ruehl M., Nakamura T., Riecken E.-O. (1998). Collagens in the Liver Extracellular Matrix Bind Hepatocyte Growth Factor. Gastroenterology.

[28] Mooradian D.L., Lucas R.C., Weatherbee J.A., Furcht L.T. (1989). Transforming growth factor-β1 binds to immobilized fibronectin. J. Cell. Biochem..

[29] Rahman S., Patel Y., Murray J., Patel K.V., Sumathipala R., Sobel M., Wijelath E.S. (2005). Novel hepatocyte growth factor (HGF) binding domains on fibronectin and vitronectin coordinate a distinct and amplified Met-integrin induced signalling pathway in endothelial cells. BMC Cell Biol..

[30] Schultz G.S., Wysocki A. (2008). Interactions between extracellular matrix and growth factors in wound healing. Wound Rep. Reg..

[31] Martino M.M., Hubbell J.A. (2010). The 12th–14th type III repeats of fibronectin function as a highly promiscuous growth factor-binding domain. FASEB J..

[32] Lin F., Zhu J., Tonnesen M.G., Taira B.R., McClain S.A., Singer A.J., Clark R.A.F. (2014). Fibronectin peptides that bind PDGF-BB enhance survival of cells and tissue under stress. J. Investig. Dermatol..

[33] Brizzi M.F., Tarone G., Defilippi P. (2012). Extracellular matrix, integrins, and growth factors as tailors of the stem cell niche. Curr. Opin. Cell Biol..

[34] Li W., Fan J., Chen M., Guan S., Sawcer D., Bokoch G.M., Woodley D.T. (2004). Mechanism of Human Dermal Fibroblast Migration Driven by Type I Collagen and Platelet-derived Growth Factor-BB. Mol. Biol. Cell.

[35] Jester J.V., Huang J., Petroll W.M., Cavanagh H.D. (2002). TGFβ induced myofibroblast differentiation of rabbit keratocytes requires synergistic TGFβ, PDGF and integrin signaling. Exp. Eye Res..

[36] Lam K.H., Kivanany P.B., Grose K., Yonet-Tanyeri N., Alsmadi N., Varner V.D., Petroll W.M., Schmidtke D.W. (2019). A high-throughput microfluidic method for fabricating aligned collagen fibrils to study Keratocyte behavior. Biomed. Microdevices.

[37] Saeidi N., Sander E.A., Zareian R., Ruberti J.W. (2011). Production of highly aligned collagen lamellae by combining shear force and thin film confinement. Acta Biomater..

[38] Kivanany P., Grose K., Yonet-Tanyeri N., Manohar S., Sunkara Y., Lam K., Schmidtke D., Varner V., Petroll W. (2018). An In Vitro Model for Assessing Corneal Keratocyte Spreading and Migration on Aligned Fibrillar Collagen. J. Funct. Biomater..

[39] Lam K.H. (2020). Development of Novel Aligned Collagen Fibril Coated Substrates for Mimicking Corneal Tissue Structures. Ph.D. Thesis.

[40] Di Lullo G.A., Sweeney S.M., Körkkö J., Ala-Kokko L., San Antonio J.D. (2002). Mapping the ligand-binding sites and disease-associated mutations on the most abundant protein in the human, type I collagen. J. Biol. Chem..

[41] Jester J.V., Barry P.A., Lind G.J., Petroll W.M., Garana R., Cavanagh H.D. (1994). Corneal keratocytes: In situ and in vitro organization of cytoskeletal contractile proteins. Investig. Ophthalmol. Vis. Sci..

[42] Yam G.H.F., Yusoff N.Z.B.M., Kadaba A., Tian D., Myint H.H., Beuerman R.W., Zhou L., Mehta J.S. (2015). Ex vivo propagation of human corneal stromal “Activated Keratocytes” for tissue engineering. Cell Transplant..

[43] Jester J.V., Huang J., Fisher S., Spiekerman J., Chang J.H., Wright W.E., Shay J.W. (2002). Myofibroblast Differentiation of Normal Human Keratocytes and hTERT, Extended-Life. Investig. Ophthalmol. Vis. Sci..

[44] Papers J.B.C., Plow E.F., Haas T.A., Zhang L., Loftus J., Smith J.W., Red A., Holland C. (2000). Ligand Binding to Integrins. J. Biol. Chem..

[45] Schmidinger G., Hanselmayer G., Pieh S., Lackner B., Kaminski S., Ruhswurm I., Skorpik C. (2003). Effect of tenascin and fibronectin on the migration of human corneal fibroblasts. J. Cataract Refract. Surg..

[46] To W.S., Midwood K.S. (2011). Plasma and cellular fibronectin: Distinct and independent functions during tissue repair. Fibrogenesis Tissue Repair.

[47] Watanabe K., Nakagawa S., Nishida T. (1988). Chemotactic and haptotactic activities of fibronectin for cultered rabbit corneal epithelial cells. Investig. Ophthalmol. Vis. Sci..

[48] Thannickal V.J., Lee D.Y., White E.S., Cui Z., Larios J.M., Chacon R., Horowitz J.C., Day R.M., Thomas P.E. (2003). Myofibroblast differentiation by TGF-beta 1 is dependent on cell adhesion and integrin signaling via focal adhesion kinase. J. Biol. Chem..

[49] de Jong-Hesse Y., Kampmeier J., Lang G.K., Lang G.E. (2005). Effect of extracellular matrix on proliferation and differentiation of porcine lens epithelial cells. Graefe’s Arch. Clin. Exp. Ophthalmol..

[50] Gamulescu M.A., Chen Y., He S., Spee C., Jin M., Ryan S.J., Hinton D.R. (2006). Transforming growth factor β2-induced myofibroblastic differentiation of human retinal pigment epithelial cells: Regulation by extracellular matrix proteins and hepatocyte growth factor. Exp. Eye Res..

[51] Muthusubramaniam L., Peng L., Zaitseva T., Paukshto M., Martin G.R., Desai T.A. (2012). Collagen fibril diameter and alignment promote the quiescent keratocyte phenotype. J. Biomed. Mater. Res. Part A.

[52] Phu D., Wray L.S., Warren R.V., Haskell R.C., Orwin E.J. (2011). Effect of Substrate Composition and Alignment on Corneal Cell Phenotype. Tissue Eng. Part A.

[53] Wray L.S., Orwin E.J. (2009). Recreating the Microenvironment of the Native Cornea for Tissue Engineering Applications. Tissue Eng. Part A.

[54] Haber M., Cao Z., Panjwani N., Bedenice D., Li W.W., Provost P.J. (2003). Effects of growth factors (EGF, PDGF-BB and TGF-β1) on cultured equine epithelial cells and keratocytes: Implications for wound healing. Vet. Ophthalmol..

[55] Kaur H., Chaurasia S.S., de Medeiros F.W., Agrawal V., Salomao M.Q., Singh N., Ambati B.K., Wilson S.E. (2009). Corneal stroma PDGF blockade and myofibroblast development. Exp. Eye Res..

[56] Gallego-Muñoz P., Ibares-Frías L., Valsero-Blanco M.C., Cantalapiedra-Rodriguez R., Merayo-Lloves J., Martinez-Garcia M. (2017). Cytokine Effects of TGFβ1, PDGF-BB, and bFGF, on human corneal fibroblasts proliferation and differentiation during stromal repair. Cytokine.

