# Supplementary material for: Effects of Topography and PDGF on the Response of Corneal Keratocytes to Fibronectin-Coated Surfaces
Source: J Funct Biomater. 2023 Apr 13;14(4):217. doi: 10.3390/jfb14040217 (PMC10144166; doi:10.3390/jfb14040217)
Supplement: Supplementary file 1 [file jfb-14-00217-s001.zip › jfb-2215717-supplementary.pdf]

Supplementary Data

for

Effects of Topography and PDGF on the Response of Corneal Keratocytes to Fibronectin coated Surfaces

Kevin H. Lam [1], Tarik Z. Shihabeddin [1], Jacob Awkal [1], Alex Najjar [1], Miguel Miron-Mendoza [2], Daniel P. Maruri[1], Victor D. Varner [1,4], W. Matthew Petroll [2,3], David W. Schmidtke [1,4,*]

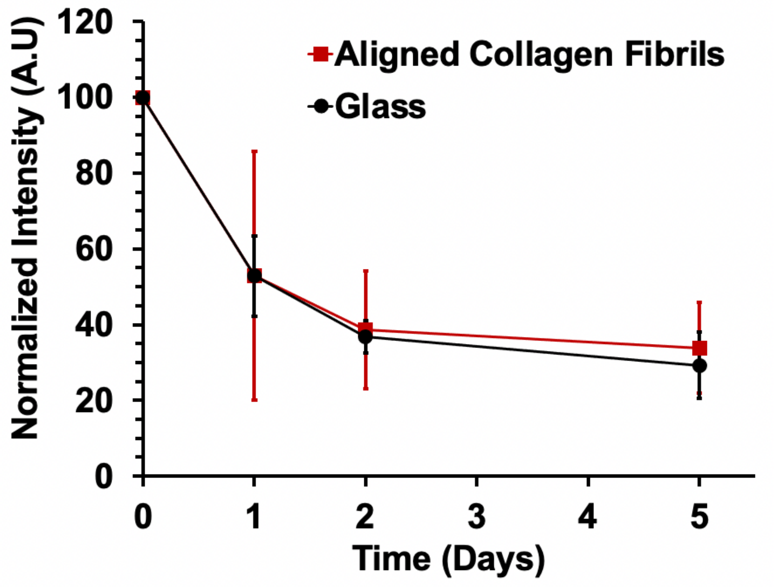

Supplementary Figure S1: Stability of the fluorescent fibronectin coating. Plot of the normalized fluorescent intensities of adsorbed fibronectin as a function of incubation times, when aligned collagen fibrils and aquasil-coated glass were both coated with a 10 μg/mL HiLyte FluorTM 488 labeled fibronectin solution (n = 3).

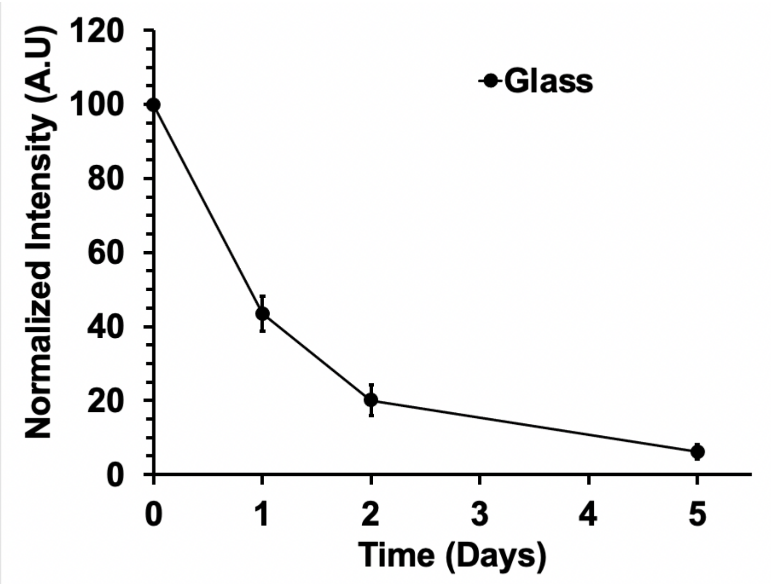

Supplementary Figure S2: Stability of the non-fluorescent fibronectin coating. Plot of the normalized fluorescent intensities of adsorbed fibronectin as a function of incubation times, when aquasil-coated glass was coated with a 10 μg/mL fibronectin solution, immunostained with a primary antibody to fibronectin, and an Alexa-647 labeled secondary antibody (n = 2).

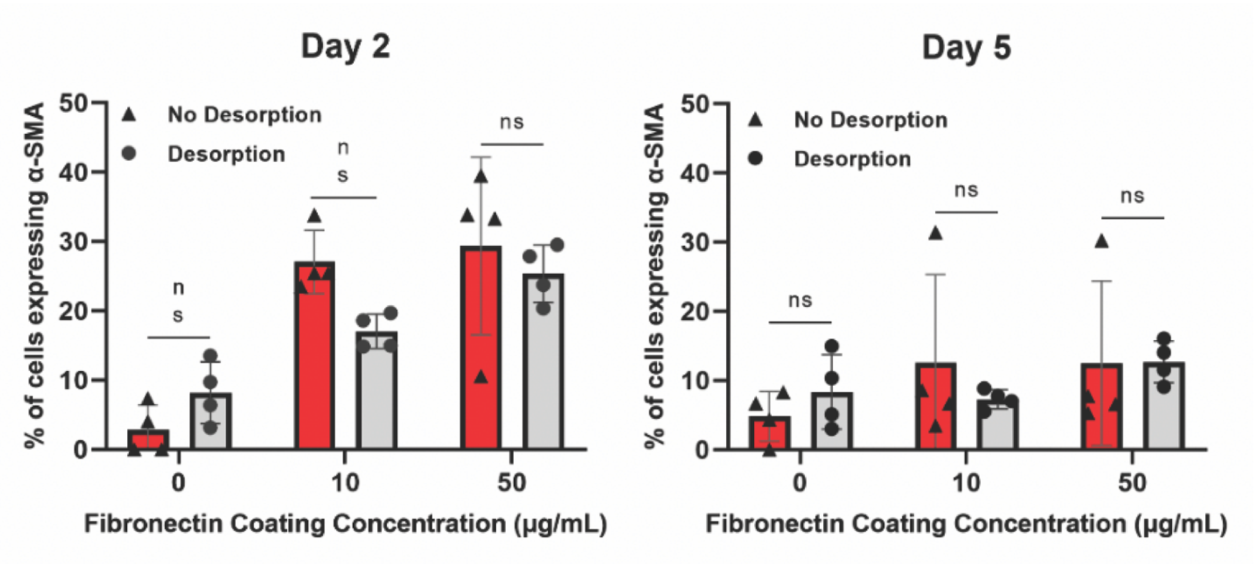

Supplementary Figure S3: Keratocyte deactivation with time on preconditioned fibronectin coated substrates. Comparison of the percentage of cells expressing α-SMA when cultured for (A) 2 days and (B) 5 days on fibronectin coated substrates with ("desorption") and without ("no desorption") preconditioning in serum-free cell culture media for 48 hrs at 37°C. Mean ± s.d. for 4 experimental replicates. A two-way ANOVA with a Tukey post-hoc test was used to evaluate significance between groups.

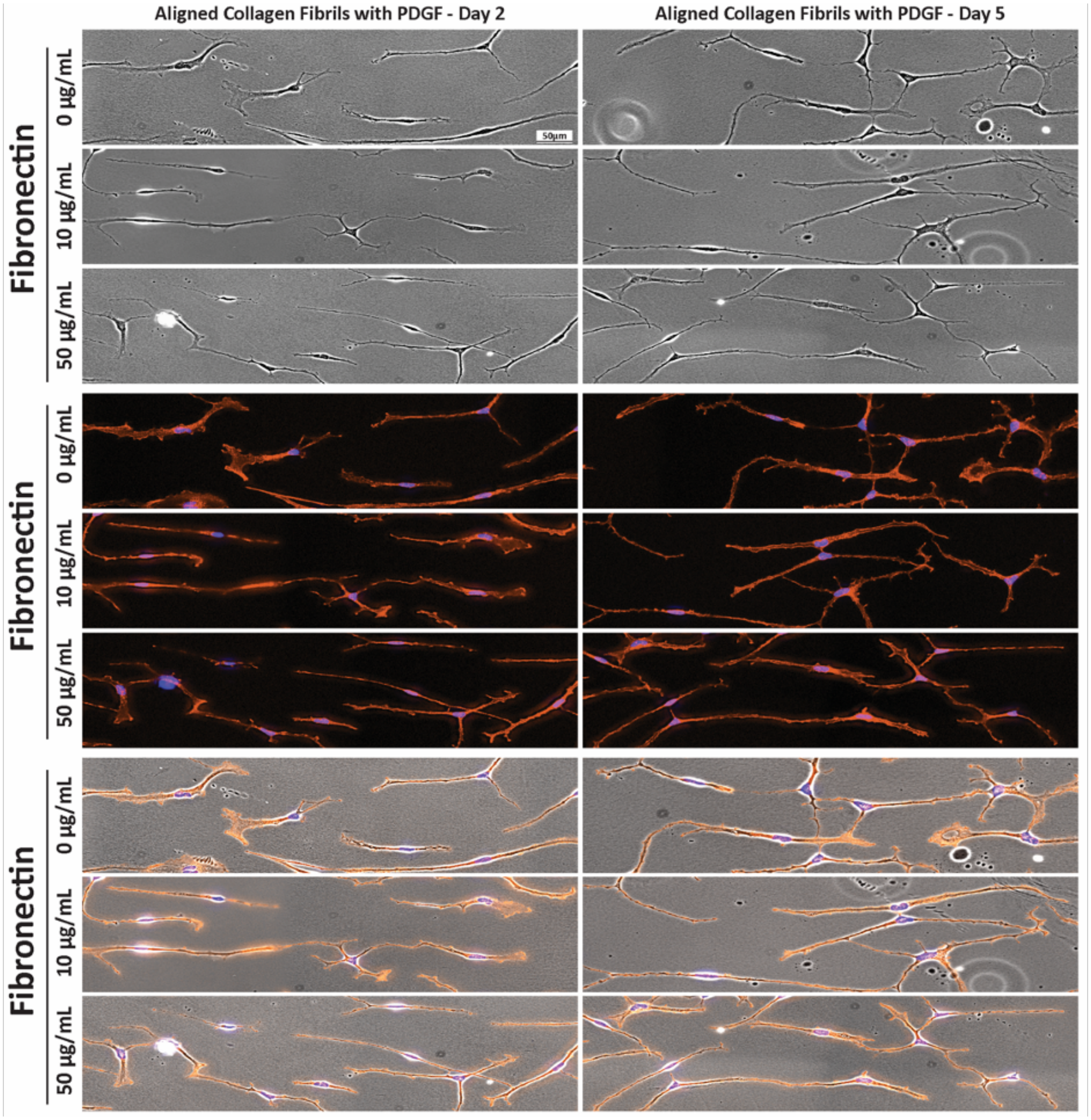

Supplementary Figure S4: Comparison of fluorescent and phase contrast images in evaluating keratocyte shape. Representative phase contrast and fluorescent images of keratocytes cultured for 2 or 5 days in the presence of 50 ng/mL PDGF-BB in solution on aligned collagen fibrils coated with different concentrations of fibronectin. Cells were stained for F-Actin (orange) and Nucleus (DAPI), scale = 100 µm. The aligned collagen fibrils were patterned horizontally.

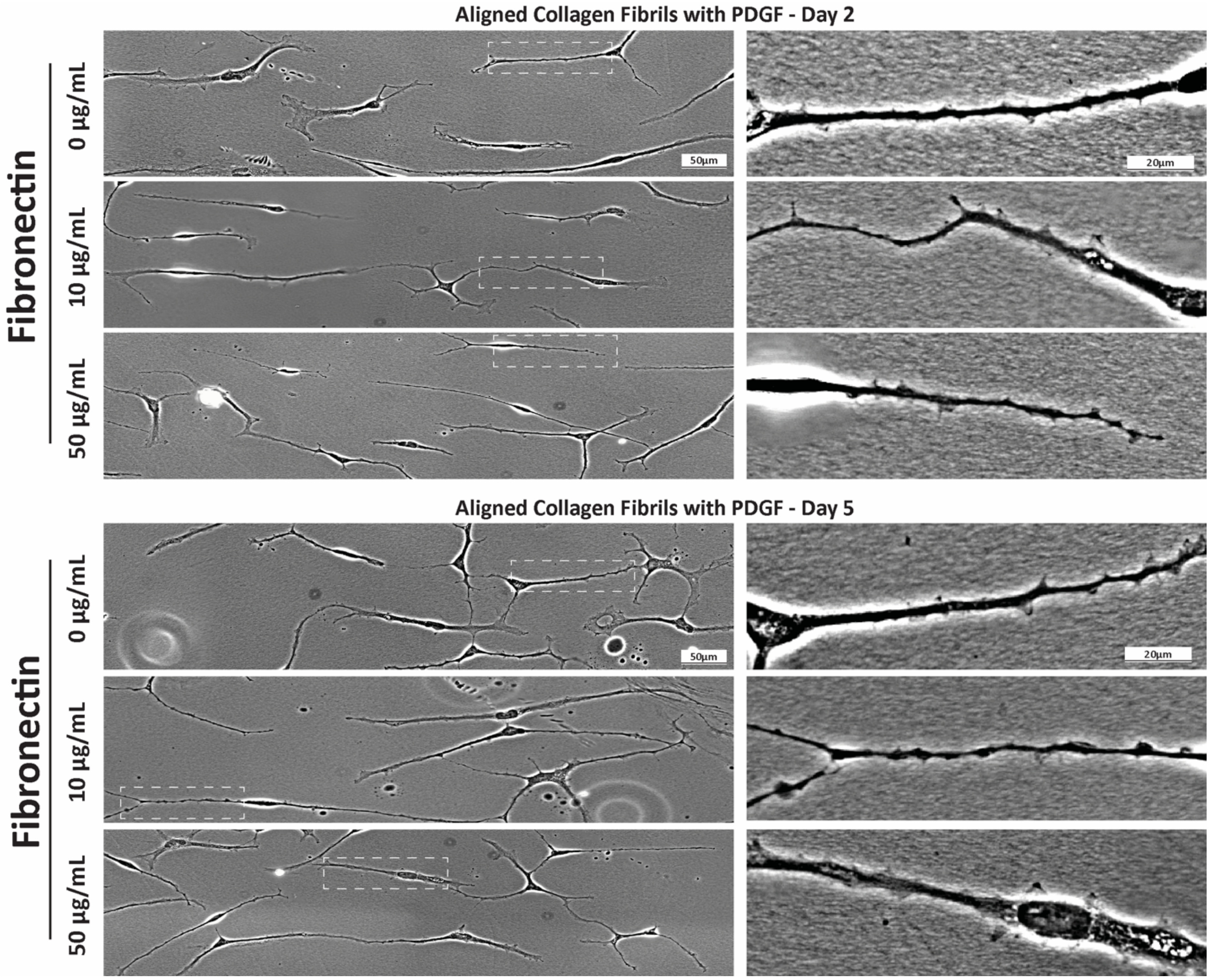

Supplementary Figure S5: Phase contrast images of keratocyte and collagen fibril alignment. Representative phase contrast images of keratocytes cultured for 2 days and 5 days in the presence of 50 ng/mL PDGF-BB in solution on aligned collagen fibrils coated with different concentrations of fibronectin. The dashed lines indicate the image inset shown to the right of each figure. The aligned collagen fibrils were patterned horizontally.
